# The aporetic dialogs of Modena on gender differences: Is it all about testosterone? Episode III: Mathematics

**DOI:** 10.1111/andr.70104

**Published:** 2025-07-28

**Authors:** Giulia Brigante, Francesco Costantino, Alessio Bellelli, Stefano Boni, Chiara Furini, Rita Cucchiara, Manuela Simoni

**Affiliations:** ^1^ Unit of Endocrinology, Department of Biomedical Metabolic and Neural Sciences University of Modena and Reggio Emilia Modena Italy; ^2^ Unit of Endocrinology Department of Medical Specialties Azienda Ospedaliero‐Universitaria of Modena Modena Italy; ^3^ Department of Engineering “Enzo Ferrari” University of Modena and Reggio Emilia Modena Italy

**Keywords:** gender, gender‐related stereotypes, hypogonadism, logic, mathematics, testosterone

## Abstract

This report is the transcript of what was discussed in a convention at the Endocrinology Unit in Modena, Italy, in the form of the aporetic dialogs of ancient Greece. It is the third episode of a series of four discussions on the differences between males and females, with a multidisciplinary approach. In this work, the role of testosterone in gender differences in the aptitude for mathematics is explored. First, the definitions of mathematical abilities were provided together with any gender difference in the distribution of females and males in science, technology, engineering, and mathematics subjects. A clear predominance of males is evident at most science, technology, engineering, and mathematics education levels, especially in advanced academic careers. Then, the discussants were divided into two groups: group 1, which illustrated the thesis that testosterone promotes the development of logical‒mathematical skills, and group 2, which, in contrast, asserted the inconsistency of a direct role of testosterone in improving cognitive abilities and that socio‐cultural factors should be considered on the basis of this gender gap. In the end, an expert referee (a female engineer) tried to resolve the aporia: are the two theories equivalent or is one superior?

## 1 INTRODUCTION

Differences between genders are evident in many biological phenomena as well as in several life aspects. The “Modena aporetic dialogs” are scientific discussions aimed at exploring the role of the hormone testosterone in determining these gender differences, if any. This episode addresses the topic of possible differences between males and females in mathematical abilities, similar to what performed for crime,^1^ empathy,^2^ and love.

It is common belief that males have a greater predisposition to study scientific rather than humanistic subjects. However, in current but also past times many women stand out for their scientific/mathematical abilities. And more and more women are enrolling in scientific degree courses—for example, medicine—obtaining excellent results. However, looking at how many women advance in academic and non‐academic careers in the scientific field, there is a striking gender gap in favor of male progression.

This manuscript reports the exchange of opinions regarding the causes of the belief in greater male ability in mathematics, starting from the data that support or undermine this idea. Furthermore, the causes of any gender differences are discussed, with particular attention to the role of testosterone, but not only.

## 2 METHODOLOGY

The discussion was carried out according to the scheme of the Socratic aporetic dialog: the question of the definition or explanation of a phenomenon is posed and then discussed, without necessarily reaching a truth. *Aporetic* dialogs typically end in aporia, a state of puzzlement by way of the equality of opposite reasonings. The discussants could discover that the opposing reasons only apparently balance each other and therefore identify the most promising theory, or that the two theses are actually equivalent, so the investigation must take a new turn or stop.

Chiara Furini (CF) provided an initial summary of the definitions of mathematical abilities. Then, she reported some data to determine if a real gender difference in these skills exists. Stefano Boni (SB—group 1) showed studies in favor of the influence of testosterone on mathematical abilities, underlining their methodological strengths and limitations. Then, Francesco Costantino (FC—group 2) has brought reasons to dismantle the cause‒effect relationship between testosterone and cognitive abilities, citing studies on both hypo‐ and hypergonadic males. Finally, Alessio Bellelli (AB—group 2) exposed other possible socio‐cultural factors that may have contributed to the belief that males are more skilled in mathematics, starting from stereotyped thinking, both individual and collective. Rita Cucchiara (RC), professor of Computer Engineering and Science, had the role of commenting on the data and, in the end, deciding whether testosterone has a role in determining any possible gender difference.

The discussion was also open to questions or comments from the audience, which included endocrinologists, biologists, biotechnologists, and residents from the Endocrinology Unit, as well as physicians in training from the School of Psychiatry of Modena (audience).

The organization of the dialog and the collection of the data presented were managed and supervised by Giulia Brigante (GB), based on an idea by Manuela Simoni (MS).

## 3 APORETIC DIALOGUE

*CF*: Before delving into any gender differences in mathematical abilities, it is useful to define what “mathematical abilities” actually mean. These are commonly described as a set of skills, both innate and acquired, including subitizing, estimation, symbolic representation of quantities, mental number line, enumeration, and arithmetic skills.

**FIGURE 1:**
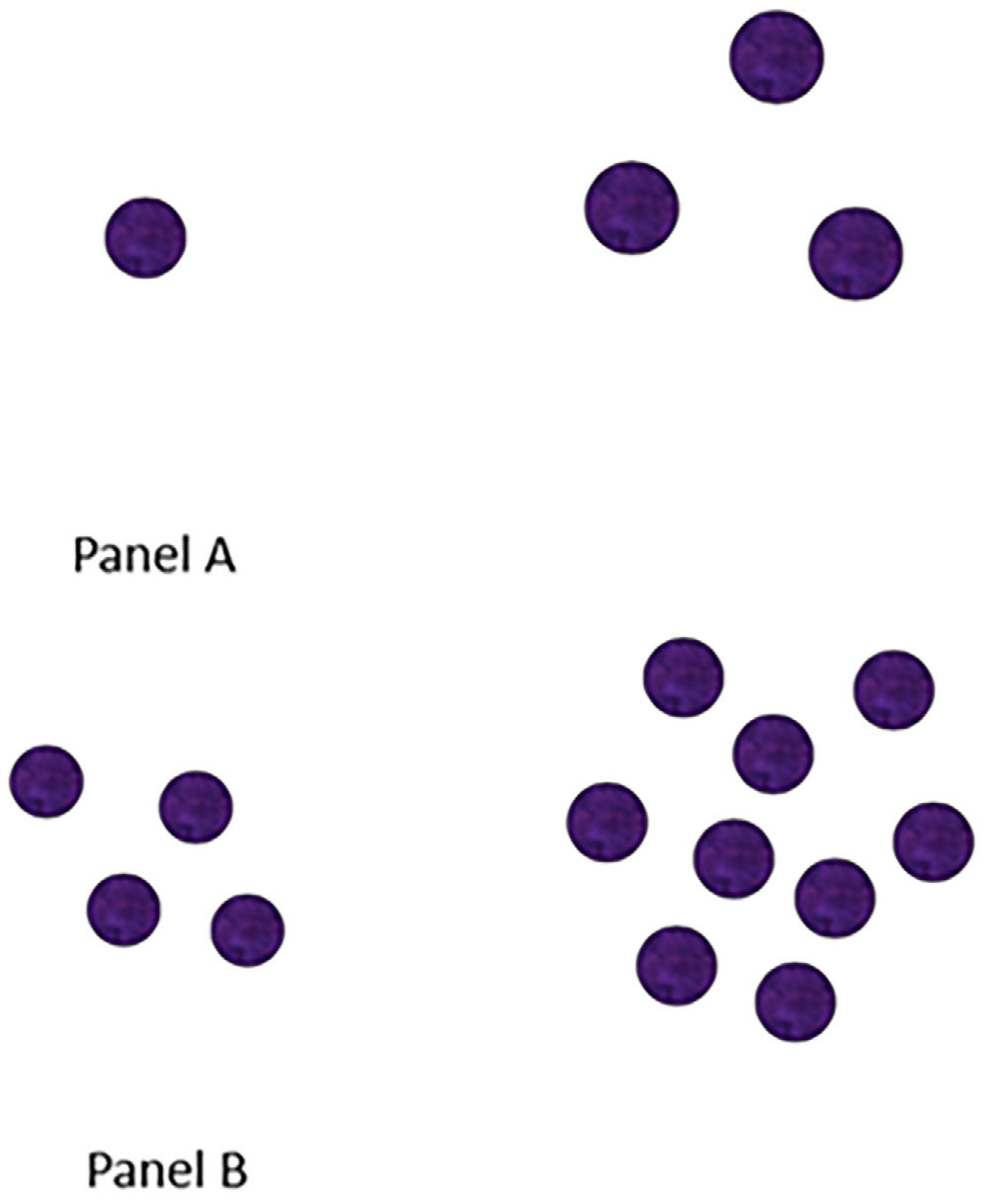
(A) Number of objects that can be counted at a glance (subitizing). (B) Larger number of objects requiring an estimate (estimation).

Subitizing is the ability to quickly and accurately identify the number of elements in a set. It is an innate capacity that enables individuals to instantly recognize a quantity at a glance without counting. As shown in Figure 1A, when fewer than three dots are present, we can instantly discern how many there are.^3^ In contrast, to estimate the number of dots in Figure 1B, we rely on a different skill: estimation. This latter allows us to make a rapid and approximate assessment of the number of elements in a set.^4^

Historically, humans invented numbers as symbolic representations of quantities, that is, the use of arbitrary and conventional symbols to precisely enumerate the number of elements of a set. The concept of the mental number line refers to the spatial representation of numbers and evidence shows that when we visualize numbers, we typically imagine them positioned along a line.^5^, ^6^ In Western cultures, this line is oriented from left to right, ranging from smaller to larger quantities. Some authors argue that this orientation is influenced by the reading direction itself.^5^, ^6^

Enumeration, a key component of counting, involves the action of passing through each element of a set exactly once, in an ordered manner, selecting the elements consecutively while retaining memory of the previous selections until the final element is reached. From this definition, we can infer the critical role of spatial representation in developing mathematical skills.

Finally, arithmetic skills include the abilities to count and perform mathematical operations. According to an evolutionary model, mathematical abilities are acquired at specific stages of life. Some, such as subitizing and numerical magnitude comparison, are innate and present from birth.^7^ During the preschool years, children acquire the verbal number system that enables counting. In the school‐age years, they learn written calculations and develop an understanding of the Arabic numeral system. Only in adulthood, according to some models, the ability to represent the mental number line become fully acquired.^7^

At this point, the question arises: Who performs better in mathematics? A useful starting point is the analysis of the winners of the Italian Mathematics Olympics. In recent years, only four women won out of a total of 120 participants.^8^

**FIGURE 2:**
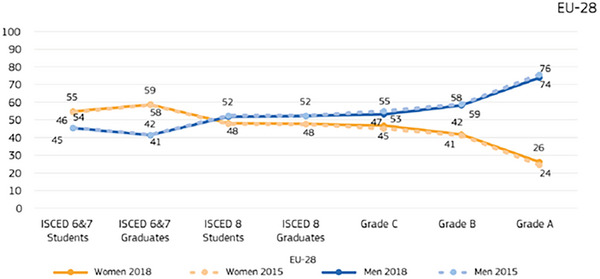
Proportion (%) of men and women in a regular academic career, students, and academic team, Eurostat EU‐28, 2015–2018.^10^ ISCED: International Standard Classification of Education.

Another notable aspect concerns gender‐based academic choices. Specifically, we can consider the number of Italian students who selected science, technology, engineering, and mathematics (STEM) subjects for the year 2023/2024.^9^ There is a clear predominance of male students, especially in computer science and technology (7600 males vs. 1500 females) and in industrial and information engineering (29,400 males vs. 9900 females).^9^ Even more noteworthy is the academic career progression of European women in STEM fields.^10^ While women consistently represent over 50% of the student population during university studies, they remain significantly underrepresented in senior academic positions. This is illustrated by the so‐called “scissors trend” shown in Figure 2,^9^ which describes the divergence between male and female representation as careers advance.

**FIGURE 3:**
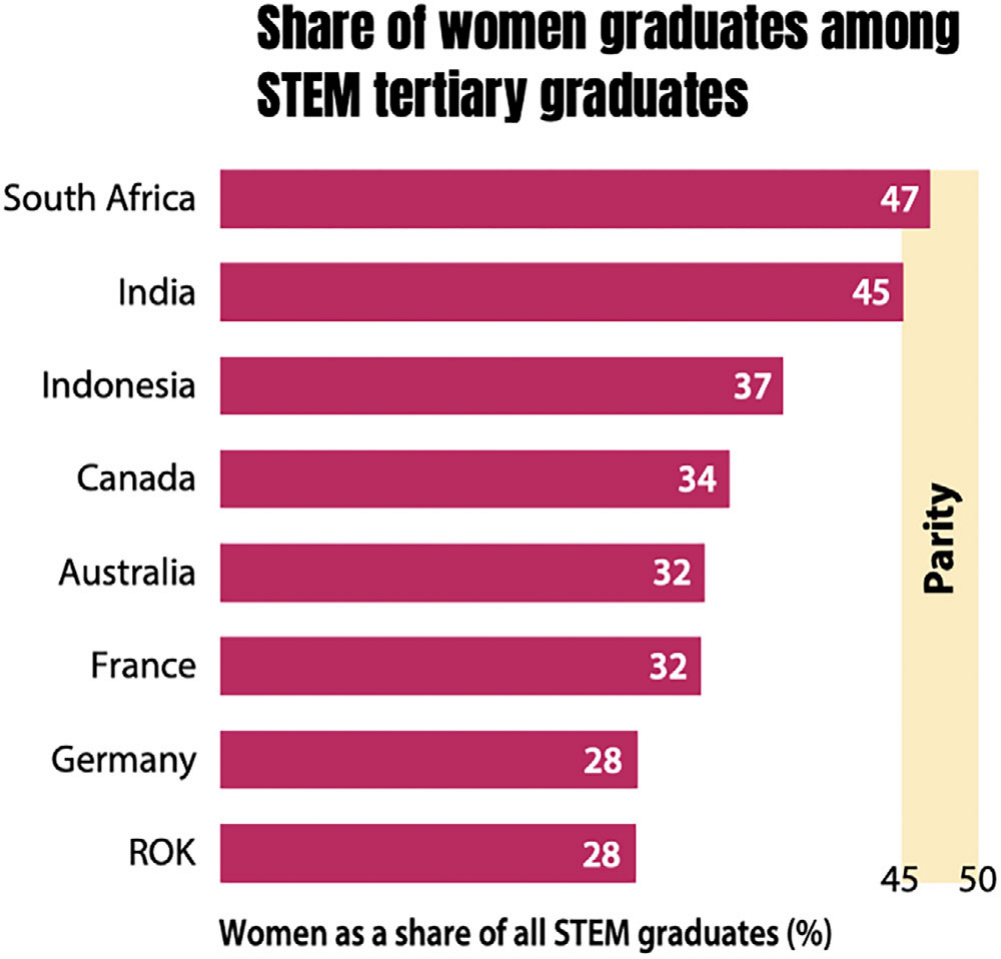
*Source*: UNESCO Institute for Statistics. 2022 or most recent data. Reproduced from UNESCO, Global Education Monitoring Report: Gender Report—Technology on her terms (2024).^11^, ^12^

Analyzing global data, while fewer girls than boys are excluded from primary education, a gender gap persists by the end of secondary education. Over the past decade, a larger proportion of men have continued to pursue STEM fields. In terms of STEM graduates, women account for only 35% of all STEM degree holders across the 18 G20 countries with available data. Notably, no G20 country has achieved gender parity in STEM disciplines, with the exceptions of South Africa (where women represent 47% of STEM graduates) and India (45%) (Figure 3). These findings highlight significant geographical disparities in the gender distribution of STEM fields.^11^, ^12^

These data suggest that men are more inclined to pursue mathematically oriented fields, whereas women tend to opt for the humanities and professions involving greater social interaction, whereas women tend to opt for the humanities and professions involving greater social interaction. However, available evidence indicates that differences are scanty in basic mathematical tasks across developmental stages during childhood, as stated by large‐scale meta‐analyses^13^, ^14^, ^15^ supporting that boys and girls perform similarly.

What accounts for this discrepancy? One possible hypothesis is the existence of inherent biological differences, such as brain structure or hormone levels, that may influence cognitive strengths and career preferences. Alternatively, social norms and cultural expectations could play a dominant role in shaping educational and professional trajectories.

*SB (group 1)*: The data presented by CF highlight the existence of a gender gap in mathematical abilities, and I would like to explore the potential role of testosterone in contributing to this difference. To support this hypothesis, I will present a series of studies that examine the effects of androgens on arithmetic, geometric, and visuospatial skills in both physiological and pathological conditions.

First, similar to verbal skills, memory, and other higher‐order cognitive functions, arithmetic proficiency relies on the integration of information across multiple brain regions and their interconnections. The classical “arithmetic network” involves fronto‐insulo‐parietal circuits.^16^ The posterior parietal cortex and the intraparietal sulcus play a crucial role in the abstraction and processing of quantitative information. The connection of these areas with the prefrontal cortex represents the central executive network, which is fundamental for working memory processes. On the other hand, the middle temporal lobe (MTL) and angular gyrus form the so‐called learning network, which is involved in retrieving acquired arithmetic knowledge such as multiplication tables, for problem solving. Two additional important networks are the salience network, which integrates external (visual and auditory) stimuli relevant to the task and the default mode network (DMN) that processes information independently of external stimuli and regulates the efficiency of arithmetic processes.^16^ Notably, functional brain MRI studies have identified sex‐based differences in the development of these brain areas: the parahippocampal gyrus and the MTL are more represented in males, while the middle and inferior frontal lobes and DMN components are more developed in females.^17^, ^18^, ^19^

This leads to a critical question: Are these sex‐related differences in brain anatomy and function mediated by testosterone? Kim et al. compared a group of 12 individuals assigned female at birth (AFAB), who had undergone gender transition from female to male and were in treatment with testosterone, with a control group of 16 fertile women.^20^ All participants underwent brain MRI to assess differences in white matter volume. Compared with controls, AFAB individuals exhibited a significantly increased white matter volume in the inferior parietal lobe (IPL), postcentral gyrus, and MTL. Furthermore, free testosterone levels in the AFAB group were positively correlated with white matter volume in both the MTL and IPL—brain areas typically more developed in males and implicated in mathematical processing. These findings suggest that testosterone may play a role in modifying brain structures associated with mathematical abilities, thereby influencing cognitive performance in this domain.

*RC (referee)*: In computer science and artificial intelligence, there is a general skepticism toward validating results derived from studies involving a limited number of participants. This represents a limitation of Kim et al.’s study.^20^ Larger studies would make it possible to adjust for variables such as age, ethnicity, and other potential confounders that cannot be adequately assessed in such small cohorts.

*SB (group 1)*: I agree. Formulating conclusions based solely on a small‐scale study is neither methodologically sound nor scientifically robust. Nevertheless, such studies can offer valuable preliminary insights into the topic. In this instance, it is noteworthy that both Kim et al. and Cheng et al. reported consistent findings.^6^, ^10^ Clearly, further investigations with larger sample sizes are warranted, but these preliminary results, in my view, are worth citing and discussing.

*Audience*: I suggest considering also ethnicity. For example, Afro‐American individuals have higher average testosterone levels compared to individuals of Caucasian or Asian ethnicity.^21^ If testosterone indeed influences these cognitive abilities, a higher mathematical performance in those groups should be expected.

*SB (group 1)*: I have not identified any study in the literature supporting this hypothesis. However, certain aspects related to ethnicity will be addressed by my colleagues.

In any case, given that testosterone appears to exert a trophic function on specific brain regions in adults, it is reasonable to question whether androgens might also influence fetal brain development.

Several hypotheses have been proposed on the mechanisms underlying the influence of androgens on brain parenchyma. Currently, it is widely accepted that testosterone strongly affects development of specific brain areas and neural circuits. This concept was initially introduced in the 1980s and 1990s through the Geschwind–Behan–Galaburda (GBG) model and the callosal.^22^

The GBG model holds that fetal exposure to high levels of androgens inhibits the neuronal migration of neural crest cells to the cerebral cortex, leading to a delayed development of left hemisphere and promoting the cognitive functions related to the right hemisphere (phenomenon known as “brain lateralization”).^23^

The callosal hypothesis claims that high levels of fetal testosterone may inhibit the pruning of corpus callosum cells, also favoring the brain lateralization.^22^ To date, several studies have shown that testosterone has a significant role in the development of both cerebral hemispheres and that it may be responsible for gender differences in a variety of behaviors, emotion processing, and cognitive functions.^24^, ^25^

The influence of testosterone on brain development has prompted further investigation into its role in the etiopathogenesis of autism spectrum disorders (ASD). ASD refers to a group of neuropsychiatric that exhibit a marked gender difference in prevalence, with a male‐to‐female ratio of 4:1.^26^

Patients with ASD are characterized by deficits in social functioning and stereotyped interests,^27^ sometimes leading to exceptional abilities in specific areas, particularly in mathematics, logic, and computer science.^28^, ^29^

In 2002, Baron‐Cohen proposed the “extreme male brain theory” as an etiological hypothesis of ASD.^30^ According to this theory, the human brain can be categorized into predominantly male and female cognitive traits. Predominantly or extremely male brains are characterized by high systematization ability—the capacity to analyze and construct systems—and low empathy—the ability to understand and share the others' feelings. Conversely, female brains are characterized by high empathy and low systematization ability. According to Baron‐Cohen's hypothesis, individuals with ASD (particularly those with Asperger syndrome [AS] and high‐functioning autism [HFA]) fall into the category of the extreme male brain.

Although this theory does not provide a comprehensive explanation for the etiology of ASD, it has the foundation for subsequent research and has stimulated investigations into the role of sex hormones in individuals with HFA or AS. Schwarz et al.^31^ evaluated various serum markers in patients with AS. Their results indicated that females with AS have higher free androgen index (testosterone/sex hormone binding globulin (SHBG) ratio) and luteinizing hormone (LH) levels compared to a healthy female control group. In males, non‐significant results were found.

Moreover, Baron‐Cohen et al. conducted a study to investigate the use of a questionnaire (autism‐spectrum quotient [AQ]) to identify the presence of “autistic traits” among adults with normal IQ.^32^ The participants were divided into four groups: 58 patients with AS or HFA, 840 students from both scientific and humanistic faculties at the University of Cambridge, 16 male winners of the UK Mathematical Olympiad, and 174 control subjects randomly selected.^32^ The highest scores were found in AS or HFA patients, followed by the group of Mathematical Olympiad winners. These results, together with the marked gender difference in autism prevalence, support the hypothesis of gender difference underlying mathematical abilities.

*RC (referee)*: The AQ scores are very similar between males and females across all groups, so there is no significant statistical difference between genders.

*SB (group 1)*: Yes, there are no statistically significant gender differences. I presented these results in order to highlight that the scores were higher in the group of Mathematical Olympics winners compared to the other two groups.

Now, I will present the results of a study conducted on patients with congenital adrenal hyperplasia (CAH). It is an endocrinologic disease caused by the deficiency of enzymes involved in different steps of steroidogenesis, leading to increased androgen levels. Therefore, these patients are a model of androgen excess.

Puts et al. conducted a meta‐analysis on the relationship between prenatal androgen exposure and spatial abilities, considering the 2D:4D ratio as an indirect indicator of fetal androgen levels.^33^ Given the role of estradiol and testosterone in the growth of phalanges, the ratio between the length of the second (2D) and the fourth (4D) finger has been proposed as a surrogate of fetus’ androgen exposure. Indeed, males have a lower 2D:4D ratio compared to females.^34^

**FIGURE 4:**
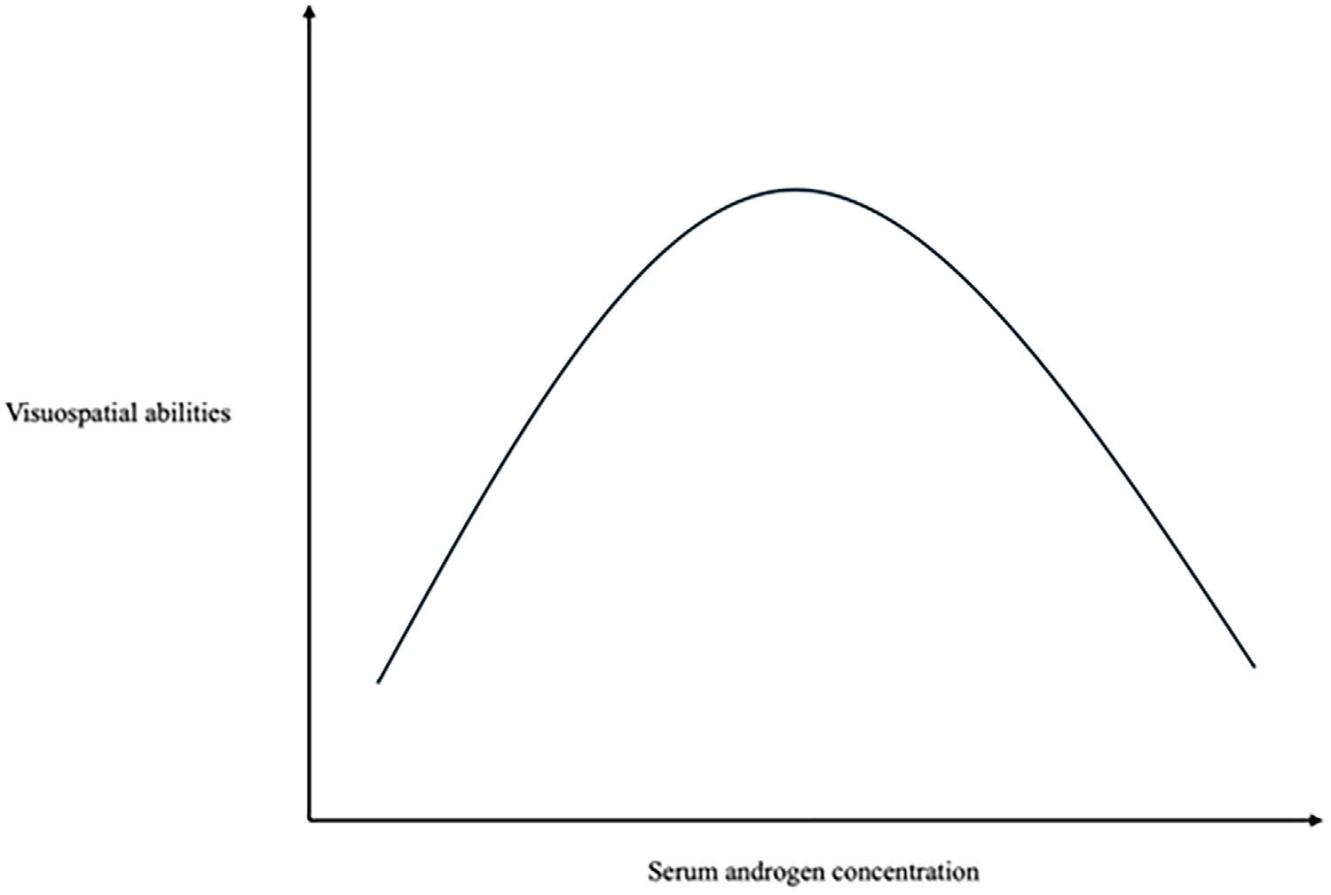
Exemplification of the relationship between androgen levels and visuospatial abilities.

Puts et al. found that females with CAH had a lower 2D:4D ratio compared to healthy females, indicating higher androgen exposure.^33^ Moreover, they had better spatial abilities compared to non‐CAH females. In contrast, males with CAH had poorer results compared to same‐sex controls. These apparently contradictory results introduce an important concept: the relationship between fetal androgen levels and visuospatial abilities is not linear, but it can be represented by an inverted U‐shaped curve (Figure 4). Both extremely low and high androgen levels are associated with poorer spatial ability.

Results consistent with the previous study were also found in a group of 516 students (304 of whom were female) from the Faculty of Economics and Business at the University of Granada. The authors measured the 2D:4D ratio in all participants and collected the grades obtained in the mathematics examination taken by the students during their first year of university. Data analysis revealed an inverted U‐shaped relationship between the 2D:4D ratio and performance in the mathematics test: students with either a lower or higher 2D:4D ratio scored worse on the test, while the highest grades were found in the group of students with an intermediate 2D:4D ratio.^35^

To investigate the gender differences in mathematical abilities in school‐aged children, I propose considering an old, but relevant study conducted by Benbow in 1988, which involved hundreds of thousands of students aged 12‒13 years in the USA.^36^ The author analyzed the results of the scholastic aptitude test (SAT),^37^ which annually assesses students' mathematical and linguistic competencies in the USA. The SAT‐M (mathematics) is the part of the test that evaluates mathematical abilities. In this study, the average scores of males were higher compared to their peers. Furthermore, within the group of highest scorers, the male‐to‐female ratio was 13:1, indicating a clear predominance of males among the top performers.^36^

*RC (referee)*: Benbow's study^36^ was conducted in American schools over 30 years ago and we cannot ignore the social bias present at that time.

*SB (group 1)*: This is certainly an important factor to consider when interpreting the results.

I would now like to present a study on adult subjects. Cherrier et al. conducted a clinical trial to investigate the short‐term effect of testosterone administration on cognitive functions in eugonadal adult men over 50 years of age.^38^ Participants were randomly assigned to two groups: 13 subjects underwent active treatment with testosterone enanthate 100 mg intramuscular once weekly for 6 weeks, and 12 controls received placebo. All participants underwent tests evaluating verbal, memory, and spatial abilities. In the domain of spatial abilities and memory, statistically significant results were obtained for two tests: the block design test and the route test. The former is a test measuring participants’ ability to analyze and construct abstract figures from their component parts. The subject is shown individual red and white cubed blocks and is asked to construct a target image by using nine three‐dimensional blocks with red and white sides. The time taken to complete the task is recorded for each attempt. The testosterone‐treated group showed a significant improvement from baseline to week 6, demonstrated by a reduction in the time needed to complete the task. Moreover, at week 6, there was a significant difference between the treated and the control group. The route test assesses the ability to navigate a short route within a room. The examiner creates a specific route on the floor using a bright red ribbon. The subject is instructed to walk the route as shown. Afterward, the ribbon is removed, and the subject is asked to retrace the route during three trials at different times. Performance is assessed by calculating the number of correct sequential units summed across all trials. In the route test, at week 3, the testosterone‐treated group showed significantly better results compared to the control group. Although this trend was not statistically significant at week 6, further supporting a role for testosterone in enhancing spatial memory.

*RC (referee)*: Did testosterone therapy lead to improvements solely in spatial abilities or were there improvements in other cognitive functions as well? Any improvement in other types of abilities or cognitive functions could explain the improvement observed in spatial abilities.

*SB (group 1)*: In addition to spatial abilities, verbal memory significantly improved after testosterone treatment.

In conclusion, there appear to be gender differences in mathematical abilities. Given testosterone's influence on the development of certain brain areas both prenatally and in adulthood and its role in visuospatial abilities, testosterone could be a key factor in explaining these observed differences.

*FC (group 2)*: The points discussed so far, coupled with epidemiological studies suggesting an indirect association between serum testosterone levels and cognitive decline during male aging, led to the assumption of a cause‒effect relationship between testosterone and cognitive abilities.^39^, ^40^ However, this theory has not been confirmed by the literature, which has examined the issue from various perspectives—namely, changes in cognitive performance following transitions from hypogonadism to eugonadism, eugonadism to hypogonadism, and in states of hyperandrogenism.

First, the most recent guidelines issued by the Endocrine Society and the Italian Society of Andrology and Sexual Medicine (SIAMS) with the Italian Society of Endocrinology^41^ advise against the use of testosterone replacement therapy in adults to enhance memory or other cognitive domains.^42^, ^43^

A pivotal reference in this context is the testosterone trial, a multicenter, placebo‐controlled, double‐blind clinical study. Eligible participants were men aged ≥65 years of age, with a mean testosterone serum level <275 ng/dL, a low risk of prostate cancer, and clinical signs and symptoms suggestive of hypogonadism (i.e., mobility limitation, diminished libido, and/or low vitality).^44^ The exclusion criteria included a Mini Mental State Examination score <24, as well as untreated or unstable major psychiatric disorders, or the need for changes in psychiatric treatment within the previous 3 months.^45^

A sub‐analysis of the project, known as the cognitive function trial, aimed to evaluate if testosterone replacement therapy would improve any aspect of cognitive function in hypogonadal men with age‐associated memory impairment (AAMI). AAMI was defined by both subjective memory complaints, which are assessed using the Memory Assessment Clinics Questionnaire, and objective memory impairment, determined through delayed paragraph recall or visual memory scores. Intriguingly, among the 493 enrolled participants, 12 months of testosterone treatment did not yield improvements in any of the cognitive domains assessed by the tests (delayed paragraph recall, visual memory, spatial ability, executive function, subjective memory complaints, global cognitive function, or immediate paragraph recall) when compared to placebo.^46^ Finally, considering all the 785 participants in the trial, the treatment group exhibited a consistent improvement in the executive function as measured by the Trail‐Making Test B minus A. However, the small effect size (adjusted estimated difference: ‒5.68 [95% confidence interval, CI ‒11.18 to ‒0.17]; *p* = 0.04) was deemed insufficient to justify androgen replacement therapy for cognitive enhancement, according to the study authors. Conversely, it is noteworthy that cognitive abilities do not appear to decline following the transition from normal to reduced testosterone levels.

A meta‐analysis encompassing two prospective studies (442 subjects) and four retrospective cohort studies (67,644 subjects) involving patients undergoing chemical castration for prostate cancer found no consistent evidence of overall cognitive deterioration in men deprived of testosterone's effects.^47^ In summary, there are no clear proofs that testosterone replacement therapy or acquired hypogonadism affect cognitive performance.

Exploring cognitive function in men with hyperandrogenism could provide further insight, but this field has been investigated rarely.

A recent observational study compared 72 weightlifters with at least 1 year of cumulative anabolic androgenic steroids self‐administration to 68 weightlifting controls.^48^ The exposed group showed an expected higher muscular strength (mean bench press record in kilograms 169 ± 31 vs. 135.4 ± 33; *p* < 0.001), but unexpectedly a reduced IQ (mean 105.6 ± 11.8 vs. 113.3 ± 9.5; *p* < 0.001). This latter finding warrants cautious interpretation. The exposed group had consistently higher self‐reported attention deficit/hyperactivity disorder (ADHD) symptom scores at the Achenbach system of empirically based assessment, which represents the most important limit. Indeed, the same study, in accordance with previous literature, found that ADHD symptoms correlate inversely with different cognitive scores such as working memory and problem solving.^49^, ^50^

To further support the notion that testosterone does not play a central biological role in enhancing cognitive functions, consider the findings of a large‐scale observational cohort study. This study analyzed data from over 7 million students who undertook the National Assessment of Educational Progress to evaluate their math performance in 10 distinct USA states. Each grade level from 2 to 11 was matched to the same grade within the same state.^51^ The weighted mean effect sizes for gender differences in math abilities across grades and states was 0.0065, indicating a negligible difference.

However, in contrast to this result, authors highlighted a slightly greater male variability in scores, with boys being more represented in the best performers (i.e., above the 95th and 99th percentiles). The reasons for this male predominance at the upper extremes remain unclear. However, the authors noted that this disparity is insufficient to account for the underrepresentation of women in fields such as engineering, where they constitute only about 15% of Ph.D. candidate.^52^ In addition, students from Minnesota shared similar data to those predicted by theoretical models and showed a male‐to‐female ratio above 95th and 99th percentiles lower in the Asian/Pacific Islander ethnicity than the one in the White ethnicity, with values of 1.09 versus 1.45 and 0.91 versus 2.06, respectively.^51^ This suggests that social and economic factors may influence mathematical performance.

*RC (referee)*: Does the last article compare other ethnicities in Minnesota regarding the male‐to‐female ratio of children scoring above the 95th and 99th percentiles?

*FC (group 2)*: The number of students scoring above the 95th percentile in other ethnic groups was insufficient to generate statistically reliable estimates. As a result, no additional comparisons between ethnicities regarding male‐to‐female ratios among top‐performing students were conducted.

*AB (group 2)*: If testosterone is not the underlying cause, other factors must contribute to the objective gender differences previously highlighted by CF. What are these contributing factors? To explore this question, I am sharing with you the results of this study,^53^ where scientists created novel, meaningless words to eliminate any possible pre‐existing gender misconception and to investigate the effect of some statements on the perception of gender stereotype. A total of 10 made up‐words were invented and used to create two kinds of sentences: the first was the so‐called *subject‒subject statement* (e.g., “Girls and boys are equally good at *freeching*”), and the second called *subject‒complement statement* (“Girls are as good as boys at *freeching*”). These sentences were presented to 288 adult participants (148 women and 140 men), who were asked which gender was more skilled in that nonsense activity. In the case of *subject–subject statements*, responses were evenly split, with approximately 50% attributing greater skill to boys and 50% to girls. On the contrary, in *subject‒complement statements*, when the statement concerns ability, participants infer that the item in the complement position (“boys”) is naturally more skilled than the item in the subject position (“girls”). Although people commonly use statements such as the subject‒complement one to express gender equality, unintentionally perpetuate the stereotype that boys are naturally more skilled and represent the benchmark to aspire to. The same experiment was repeated submitting the statements to a group of 337 7‒11‐year‐old children showing the same results. These findings indicate that this semantically rooted stereotype is already present in early childhood.

At this juncture, it is pertinent to examine the theoretical frameworks underpinning gender stereotypes in mathematics. The literature mainly reports three theories. The first is the one previously mentioned, called the *empathizing‒systemizing theory*: there is a difference between females and males because females are less exposed to prenatal testosterone and thus develop more empathy; on the other hand, males develop a more systematic thinking mode having been exposed to higher concentrations of testosterone. The second theory is called *Girls’ Compensation*: females achieve comparable mathematical performance to males not because of equal innate ability but because they exert greater effort to compensate for a presumed lack of natural talent. The third theory is called *Girls’ Non‐Compensability*: girls are not only less talented than boys in mathematics but also lack the tools to compensate.

These theories defining gender stereotypes have been investigated by various studies, including one published in 2022,^54^ using the *Math‐Gender Misconceptions Questionnaire*. The study surveyed 303 future teachers, predominantly female, 50% of them employed in STEM fields. Participants responded to statements designed to assess agreement with gender‐based misconceptions in mathematics (e.g., “Despite their on average stronger effort, girls are normally less proficient in math than boys”). Then, a misconception score was calculated. Analysis revealed that stereotypes falling under the *Empathizing‒Systemizing* and *Girls’ Compensation* theories were the most represented, in 32% and 26.7% of participants, respectively. The least represented stereotype was related to the *Girls’ Non‐Compensability* theory (17.5%). The notable aspect highlighted by the study is that about half of the participants (48.2%) had at least one gender misconception in mathematics, and 44.6% had at least two of them. These data highlight the prevalence of gender stereotypes when talking about math skills, even in future, teachers should have the task of ensuring equal training without preconceptions.

A study conducted in Italy investigated the impact of stereotype threat on primary school students' attitudes, performance, and perceptions regarding mathematics.^55^ The research involved 476 children across second, third, and fifth grades and was structured in two phases. In the initial one, researchers submitted a questionnaire to the children investigating their beliefs and opinions about mathematics (self‐confidence, perceived difficulty, gender stereotypes, value attributed to mathematics). In the second phase, with the aim of inducing the stereotype to the children and understanding if it is able to affect their mathematical performance, the researchers divided the group into two subgroups: the test group was shown 10 images of famous mathematicians worldwide, nine males and one female; the control group was shown 10 generic images, nine flowers and one fruit. After observing the images, the internalization of stereotype was ensured by asking the children how many images were female and how many were male, with different methods depending on their level of education. After this, they were given a math test to measure how much the induced stereotype influences their performance. Analyzing the results of the first phase, in terms of self‐confidence, there is no difference in the second and third grades, but it becomes statistically significant when considering the fifth grade where boys are much more confident than girls in mathematics. As for the perceived difficulty, there is no significant difference between boys and girls. Finally, researchers asked participants to assess whether boys were better than girls, girls were better than boys, or boys and girls were equally good. The interesting result is that while second grade girls considered themselves more skilled than boys and boys considered themselves neutral, in fifth grade, both boys and girls thought boys were more skilled than girls in mathematics. This also agrees with the results obtained after the primary mental ability test submitted in the second phase of the study, where fifth‐grade girls exposed to the stereotype performed worse than girls not exposed to the gender stereotype.

An analysis of the scientific field reveals that women tend to publish fewer articles and patents compared to men, a trend that constitutes a noteworthy observation. Does this mean that female scientists are less productive than male scientists? According to existing literature, women's lower scientific productivity has been attributed to several factors, including less supportive work environments, concurrent family responsibilities, differing roles within research teams, and variations in the type of supervision received.^39^ However, a study published in Nature in 2022 provides compelling evidence that the disparity in productivity may largely stem from the undervaluation of women's contributions.^56^ The authors reported that although women represented 48.2% of the scientific teams' workforce of 57 college campuses, they accounted for only 34.8% of the authors listed in published scientific articles. To confirm this, the authorship of research groups was investigated by calculating the ratio between potential authorship and actual authorship among the females employed from 2013 to 2016. They found that the proportion of actual female authors is lower than expected given their numbers among potential female authors at every career stage and in every field of research. This indicates that far fewer articles include women than would be expected, and the women involved attributed this underrepresentation to the undervaluation of their scientific contributions. It cannot be excluded that these gender differences may depend on a different composition of research teams in terms of men and women, research field, time dedicated to projects, or job title. However, this gender difference persists not only in absolute values but also when all the confounding factors mentioned before are considered. Similarly, the authors also found that there is no significant difference between the probability of a woman being cited compared to a man in an article with zero citations. On the contrary, women are less likely than men to be cited when the most cited articles are considered. This negative trend persists with regards to patents.

Further insights emerge from international assessments evaluating educational progress. A significant disparity in problem‐solving performance was observed across various countries with boys outperforming girls in most, except for the United Arab Emirates, Bulgaria, Finland, and Montenegro.^57^ Some could speculate that life‐quality pressures in less gender‐equal countries and socio‐cultural factors might have a role in such differences. Moreover, research indicates that women experience higher levels of mathematics anxiety compared to men, which correlates with reduced performance. Parental expectations also play a role, as the analyzed answers indicate that parents have stereotyped expectations: they expect their sons to work in STEM fields after completing their studies and their daughters in non‐STEM fields.

These findings underscore the impact of stereotypes on girls' academic choices and performance from an early age.

*RC (referee)*: I agree with your assessment. I have observed that women working in STEM field are much more anxious, less confident in themselves, compared to men. However, recently, especially after COVID‐19 pandemic, I have seen more and more stressed boys. I think that the situation is somehow balancing out. However, all these data highlight that important gender differences in STEM fields are not directly related to testosterone.

*MS*: I am not convinced that testosterone is to blame, for several reasons. First, as AB has shown, the bias of stereotypes is huge. Second, it is impossible to design controlled experiments in research to prove this thesis. In the past, there has been an overwhelming prevalence of the male sex, not only in the STEM field but in the entire scientific community. A well‐known case of low recognition of scientific contribution by women is that of Rosalind Franklin.

Watson and Crick received the Nobel Prize for the discovery of the DNA double helix, but Franklin's pivotal contribution initially went unrecognized. This historical oversight illustrates both the legacy we inherit and the challenges that persist today.

## 4 CONCLUSIONS

*Manuela Simoni*: Thank you for presenting such valuable data, especially considering how challenging it is to find robust literature on this important topic. To sum up, while the earlier studies and theories have suggested a potential male advantage in mathematical abilities,^20^, ^22^, ^30^, ^33^, ^36^, ^38^ more recent literature is in contrast with these findings. Indeed, the potential role of testosterone in shaping neuroarchitecture and influencing cognitive function remains inconsistent and inconclusive.^46^, ^47^, ^51^ Furthermore, I considered it relevant to address how this assumption, although scientifically unsupported, persists in public discourse and educational environments, where it may negatively impact students' self‐perception and influence career trajectories in science, technology, engineering, and mathematics fields.^54^, ^55^, ^56^

Does the referee declare a winner? Is the team that claims that testosterone makes males more skilled in mathematical‒scientific fields or the one that believes that there are other factors or even that superiority does not exist?

*Rita Cucchiara (referee)*: I would give a special mention to Chiara Furini for presenting objective, indisputable data, telling a great truth in the science, technology, engineering, and mathematics field. Regarding the debate on whether testosterone is to blame, I have to say that I found the reasoning about stereotypes more persuasive. I reckon that stereotypes play a more substantial role than testosterone—or any biological factor—in shaping the belief that females are less capable than males in mathematical and scientific disciplines.

## AUTHOR CONTRIBUTIONS

Manuela Simoni had the idea of the discussion on this topic and the aporetic dialog structure. Francesco Costantino, Alessio Bellelli, Stefano Boni, and Chiara Furini did the bibliographic research and created the data presentation under Giulia Brigante supervision. Rita Cucchiara, Manuela Simoni, Giulia Brigante, Francesco Costantino, Alessio Bellelli, Stefano Boni, and Chiara Furini discussed and commented on the data and wrote the paper. Giulia Brigante and Francesco Costantino submitted the paper.

## CONFLICT OF INTEREST STATEMENT

The authors declare no conflicts of interest.

## FUNDING INFORMATION

The authors received no specific funding for this work.
